# Supply or Demand, that is the Question: Decomposing Euro Area Inflation

**DOI:** 10.1007/s10272-022-1092-z

**Published:** 2022-12-16

**Authors:** Paolo Pasimeni

**Affiliations:** DG GROW Breydel building (BREY), European Commission, Avenue d’Auderghem 45/Oudergemselaan 45, 1040 Brussels, Belgium

**Keywords:** C83, E31, E37, L6

## Abstract

This paper studies the recent trends in inflation in the euro area and estimates to what extent the current inflationary pressures are driven by demand expansion and by supply side disruptions. First of all, consumer price inflation is particularly pronounced in goods and less so in the case of services. Energy and food are the most relevant components of rising consumer prices, accounting for almost three-quarters of total headline inflation. The fact that price pressure comes mainly from sectors with a high import content suggests that disruptions along international supply chains may play a key role. The paper then focuses on producer prices at the sectoral level and presents a methodology to decompose the rise in inflation between supply and demand impulses. It finds that, in the present context, supply factors are the main driver and account for at least 80% of the current increase in producer prices in industry and in each one of the manufacturing sectors with the highest price pressures. There are nevertheless some differences across countries. These findings imply that, if repairing supply-side problems and disruptions along supply chains is the priority, then promoting the right investment may be more urgent than cooling demand down. Country heterogeneity in the relative importance of demand and supply factors, then, may lead to different degrees of effectiveness of monetary policy and to a risk of divergences.

The recent spike in inflation spurred concerns about the overall macroeconomic outlook of the euro area (BIS, 2022; IMF, 2022; Lane, 2022; Reis, 2022; Buelens and Zdarek, 2022; Bodnar and Schuler, 2022; Moessner, 2022; Nickel et al., 2022). The peculiar unfolding of two recent shocks put pressure on macroeconomic policies to find an adequate policy mix. The exit from the pandemic is characterised by large imbalances between supply and demand, resulting from a release of pent-up demand and deep supply chain disruptions. While those were widely understood to be of a temporary nature, the occurrence of a second shock, after the Russian invasion of Ukraine, radically changed the perception of the underlying economic processes.

As trade disruptions may well become structural, some capital and trade flows will have to adjust to the new geopolitical context, and the energy shock will likely require structural actions. The overlap of various supply side shocks, some more persistent than others, may have an influence on inflation expectations and increase the pass-through to consumer prices. The production hit also has the potential to slow growth. The combination of these shocks is bringing the European economy towards the unpleasant situation of accelerating inflation and decelerating growth. Policymakers need to make difficult decisions against this backdrop.

The revisions of the macroeconomic forecasts (Figure 1) in the last five IMF World Economic Outlooks signal that the euro area is facing a very deep and unexpected supply shock in 2022, as the sharp downward revision of potential output in the past few months suggests. A plausible hypothesis is that such shock depends on severe disruptions in international supply chains and on energy prices, and that this may be the main cause of the underlying inflation pressures. This paper tries to test this hypothesis and to estimate the relative importance of demand and supply factors.

* The views expressed are the authors' alone and cannot be attributed to the European Commission. The author is grateful to Román Arjona, Christian Buelens, William Connell, Cristina Herghelegiu, Kerstin Jorna, Josefina Monteagudo and Outi Slotboom for the useful discussions on the topic and for the comments on previous versions of the paper, while remaining the sole responsible for any mistake in this document.

**Paolo Pasimeni**, European Commission; and Brussels School of Governance, Belgium.

Figure 1
**Forecasts of main indicators for 2022 in the euro area**

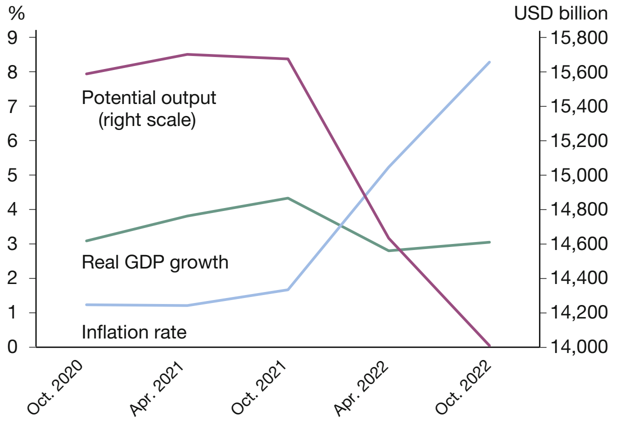

Note: The chart shows the forecasts for the year 2022 of euro area real GDP growth, inflation and potential output in the five most recent waves of the IMF World Economic Outlook.

Source: Author's elaboration on IMF data.

Figure 2
**Price level in the euro area**
Harmonised consumer price index

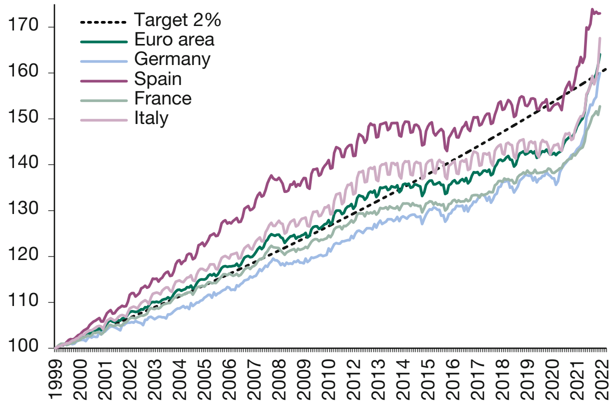

Note: Monthly data, year-on-year change, 1999=100.

Source: Own elaboration on Eurostat data.

## Consumer price levels

The recent data about consumer prices show that, after years of subdued inflation, headline inflation in the past year has been consistently and increasingly above the 2% target. The recent forecasts present continuous upward revisions of inflation, which is now projected in a range between 5.3% and 8.1% in 2022, and 2.3% and 5.5% in 2023 for the euro area. Markets expect inflation to peak in mid-2022 and then decline, but to remain elevated (ECB, 2022a). The rise in commodity prices is driving up inflation and reducing purchasing power in the absence of wage indexation. This may pose a drag on growth, by constraining consumption and consumers' confidence (ECB, 2022b).

Analysed from a long-term perspective, the recent spike in inflation has brought back the overall price level in the euro area to the price level corresponding to the 2% target, had it been maintained each year since the creation of the euro area (Figure 2). Inflation in the euro area was moving close to target until 2014, but it remained remarkably subdued in the following six years until the end of 2020. In the past year and a half, it has nevertheless increased at an unprecedented rate. While the overall price level is close to the level it would have had [1]if the target had been achieved each year, the recent acceleration poses serious concerns about the possibility to rein in inflation in the near future, without causing major harm to the economy. The country detail shows quite some heterogeneity in the inflation trends, which pose concerns for the conduct of the common monetary policy and the overall macroeconomic stability of the monetary union.[2]

## Goods vs services

One of the key characteristics of the current price dynamics is the decoupling between services and goods. While the former have kept a rather stable inflation trend, prices in the latter have been more volatile and are on a historically increasing path, now above 10% year-on-year. Figure 3 shows the monthly year-on-year change in the harmonised index of consumer prices (HICP), as well as in the main underlying expenditure categories. Core inflation[3] is less volatile and shows a more stable trend, but it is now increasing well above target. The increasing trend in prices of goods is driven in particular by industrial goods.

Specific survey data confirm this dichotomy between industry and services. In August 2022, two out of three companies operating in the service sectors in the EU still report some factor limiting their business activity production, while this share increases to three out of four in in-

1 In August and September 2022 the price level was in line with the target; in October it has overcome it.

2 For a detailed analysis of the risks associated with inflation differentials within the monetary union, see Acocella and Pasimeni (2018).

3 So-called core inflation is measured by the HICP excluding energy, food, alcohol and tobacco, i.e. the most volatile components, leaving a measure that better reflects the underlying structural conditions of the economy.

Figure 3
**Inflation trends in the euro area**
Monthly, year-on-year change

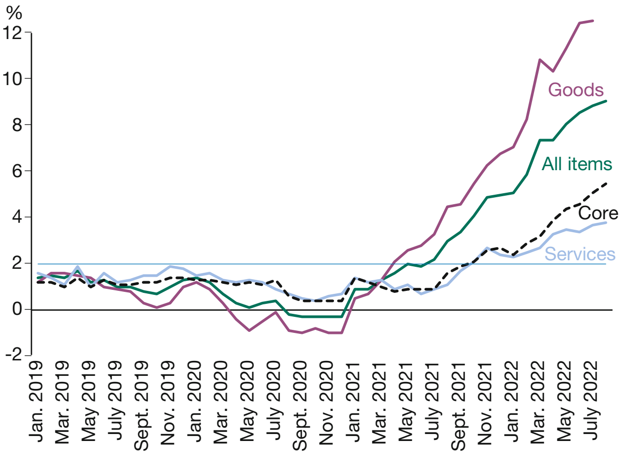

Source: Eurostat.

Figure 4
**Producer price inflation and labour intensity in the euro area at the sectoral level**

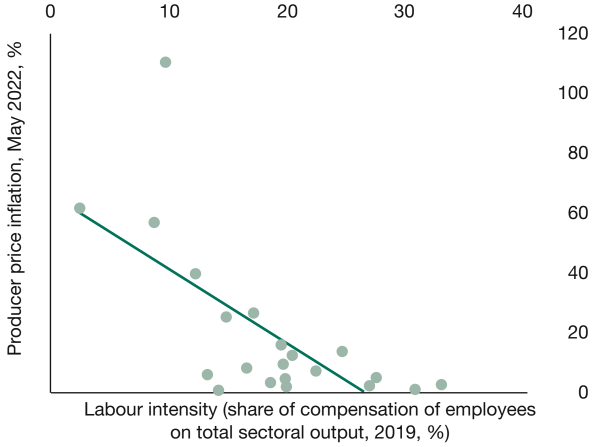

Source: Own elaboration on Eurostat data.

dustry (European Commission, 2022). It is the first time on record that so many firms report concerns about factors limiting their production in industry, and also the first time that the difference between industry and services is so unfavourable for industry.

Industry sectors recovered faster than services from the pandemic, due in part to the nature of the containment measures put in place, but seem to be suffering more in the past few quarters. A closer look at the reported factors limiting the production in industry shows that, for the first time, supply-side concerns about shortages of materials and equipment are the single most critical factor. The assessment of businesses is consistent with the evidence of supply bottlenecks in several sectors. For a long time, a lack of demand had been the most critical factor reported by businesses in the euro area; but this is no longer the case, confirming the ongoing demand expansion.

The markedly divergent behaviour of inflation in goods and services suggests a couple of reflections. Services represent the largest share of the economy, so if inflation in services is moderate, then the overall inflation trend would not be as high as goods inflation may suggest. Services are more labour-intensive sectors, therefore domestic wage developments may not be the main driver of price pressures. The sectoral detail corroborates this point, since the sectors in which inflationary pressures are higher are also those in which labour intensity is lower (see Figure 4).

This observation is in line with recent analyses showing that higher wage demands in the euro area were not followed by correspondingly higher wage deals, with automatic wage indexation being more limited. Overall, there seem to be no signs yet that a price-wage spiral has started, while second round effects may still begin if price pressures continue (Buelens and Zdarek, 2022).

It becomes therefore important to investigate to what extent the recent rise in inflation is due to specific supply disruptions in some industrial sectors, coupled with the release of large pockets of demand, pent up during the long period of containment measures.

## Imported vs domestic inflation

The decomposition of headline inflation in its main components (Figure 5) shows that soaring energy and food prices are clearly driving it up, accounting now for more than two-thirds of the overall rate. Moreover, the increase in energy prices this year is unusual compared with previous episodes since price increases have been broad-based across all fuels (World Bank, 2022), reducing the possibilities for substitution.

The ECB has recently presented a new indicator (Fröhling et al., 2022) to disentangle the relative shares of domestic inflation for the euro area that takes into account the import intensity of the items considered in the harmonised indices of consumer prices. This methodology aggregates those items with a relatively low import intensity into a “Low IMport Intensity” (LIMI) inflation indicator, which is used to complement the information provided by other traditional indicators and inform monetary policy.

Figure 5

**Contributions to annual inflation in the euro area**

Monthly, year-on-year change

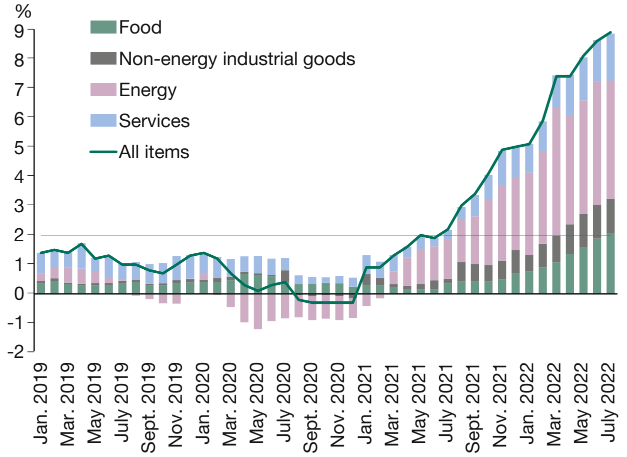

Source: Eurostat.

Figure 6

**Decomposition of inflation in import content levels**

Annual percentage changes

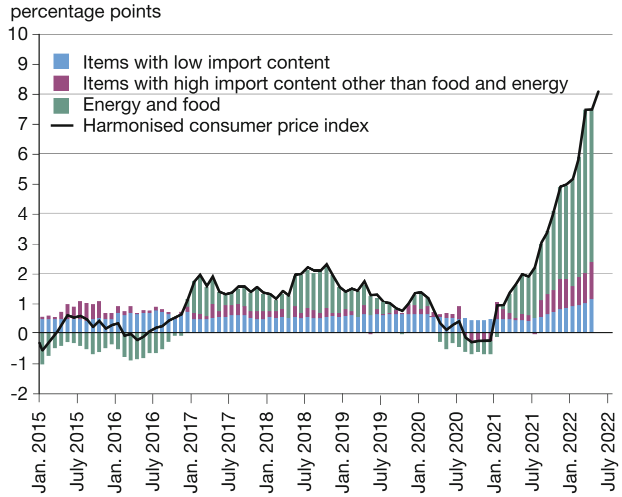

Source: Fröhling et al. (2022).

This decomposition (Figure 6) shows that beyond energy and food, accounting for the majority of price pressures, other items with high import content are now more prominent than items with low import content in contributing to inflation. Nickel et al. (2022) claim on this basis that the largest part of inflation in the euro area reflects shocks generated abroad, via net imports of energy and commodities or via the import content of other goods and services. This result adds further to the likelihood of the hypothesis that supply side disruptions, in particular along international value chains, may be the main underlying driver of inflation. In order to test this hypothesis, the next section looks at producer prices at the sectoral level.

## Supply and demand factors

This section presents a methodology to disentangle the relative contributions of supply and demand shocks to inflation. Since inflation dynamics typically depend on the changing balance between aggregate supply and aggregate demand in the economy, it may be difficult to single out sector-specific factors that drive overall inflation dynamics. We focus the analysis on producer prices in industry, which are most affected by the current price pressures.

### Data and methodology

In order to measure the relative magnitude of supply and demand shocks, we need to find some reliable and timely indicator of the changing relation between supply and demand at the sectoral level. We therefore use the assessment of demand and supply factors made by businesses themselves in the official surveys. We use actual observations collected each quarter, rather than extrapolating trends, because extrapolations of past trends to the future become inherently weak, in times of such profound disruptions. One way to measure the relative elasticity of inflation to supply and demand shocks would be through regression analysis. However, that method would provide a historical average while missing out on the specific characteristics of each single shock.

Shapiro (2022a) proposes a framework in which he decomposes inflation in the United States, using categorical-level regressions or systems of equations, and then using the estimates to group categories into components of personal consumption expenditures (PCE) inflation. In his application to the current inflation shock (Shapiro, 2022b), then, he calculates the shocks as the difference between the expected values of each component and the observed ones; then he attributes them to the relative shares of each component in total PCE.

We focus instead on producer prices at the sectoral level and use a regression analysis to establish the relevance of possible factors in explaining inflationary trends, but then opt for a different approach when it comes to measuring the relative importance of each shock in each quarter. In this case, we calculate the relative strengths of supply and demand shocks in each quarter with respect to the historical average, and decompose the total inflationary pressure into those two impulses.

To define the impulses provided by supply and demand factors to inflation, we use data provided by the quarterly survey about the main factor hampering production or business activity, which provide quite long time series.[4] When replying to this question, businesses can choose among six options, pointing to (a) no factor limiting production, (b) lack of demand, (c) shortages of material and equipment, (d) labour shortages), (e) financial constraints and (f) other.

In order to establish which of these factors are the most appropriate to signal an inflationary shock, we observe their behaviour over time in relation to price dynamics. We therefore run a set of panel regressions, by applying the following formula:

$$\pi_p = \alpha N_{jt} + \beta D_{jt} + \gamma M_{jt} + \vartheta L_{jt} + \mu F_{jt} + \rho O_{jt} + \varepsilon + \varphi + X_{jt}.$$

In this formula, the dependent variable $\pi_p$ is the year-on-year change in prices, measured each quarter for each component $p$; then the regressors are $N_{jt}$, $D_{jt}$, $M_{jt}$, $L_{jt}$, $F_{jt}$, $O_{jt}$, which represent the shares of businesses reporting, respectively, no factor limiting their production, lack of demand, shortages of materials and equipment, shortages of labour, financial constraints, and other factors, in country $j$ and in quarter $t$; $X_{jt}$ is a vector of control factors; $\varepsilon$ is the error term; and $\phi$ represents country fixed effects. $\alpha$, $\beta$, $\gamma$, $\theta$, $\mu$, and $\rho$ are the coefficients we are interested in; they will signal to what extent that factor is significantly associated with inflation dynamics.

## Inflation and factors limiting production

We first run the panel regression for several measures of inflation from the headline HICP, consumer prices of goods, consumer prices of industrial goods only, core inflation (HICP all items excluding energy and unprocessed food), producer prices and producer prices of intermediate goods only.

The results (Table 1) of this first set of panel regressions show that shortages of materials and equipment is the single most significant factor associated with inflation. In the case of producer prices in industry in particular, shortages of materials and equipment and lack of demand are the two most significant factors.

Table 1
**Price increases and factors limiting production**

| Dependent variables | HICP | Goods | Industrial goods | Core inflation | Producer prices | P.P. int. goods |
|---|---|---|---|---|---|---|
| No factor limiting production | -0.04 (.04) | 0.00 (.01) | 0.01 (.02) | 0.00 (.02) | -0.01 (.03) | 0.00 (.02) |
| Lack of demand | -0.04 (.02) | -0.02 (.02) | -0.02 (.02) | 0.00 (.02) | -0.10*** (.02) | -0.13** (.04) |
| Shortages of materials and equipment | 0.08* (.03) | 0.11*** (.02) | 0.16*** (.03) | 0.04 (.02) | 0.30*** (.06) | 0.35*** (.06) |
| Shortages of labour | -0.02 (.02) | 0.00 (.02) | -0.01 (.02) | 0.02 (.02) | 0.01 (.03) | 0.02 (.06) |
| Financial constraints | 0.04 (.03) | 0.06 (.03) | 0.09* (.04) | 0.05 (.03) | 0.05 (.04) | 0.03 (.05) |
| Other factors (unspecified) | 0.01 (.02) | 0.02 (.02) | 0.02 (.03) | 0.03 (.02) | 0.03 (.03) | 0.07* (.03) |
| Constant | 4.32* (4.32) | 1.08 (1.17) | -0.55 (1.48) | 0.80 (1.39) | 2.74 (1.69) | 2.94 (2.52) |
| Fixed effects | yes | yes | yes | yes | yes | yes |
| No. of observations | 2042 | 2027 | 2027 | 2027 | 2021 | 1999 |
| $R^2$ | 0.11 | 0.12 | 0.16 | 0.05 | 0.23 | 0.30 |
| Adjusted $R^2$ | 0.10 | 0.11 | 0.16 | 0.05 | 0.23 | 0.30 |

Note: * p<0.05, ** p<0.01, *** p<0.001. Robust standard errors in parentheses.

Source: Author's own calculation.

The first factor, as one would expect, is positively associated with inflation, meaning that when more businesses declare that they face such shortages, inflation tends to increase, and vice versa. On the other side, insufficient demand is significantly, but negatively associated with raising inflation, meaning that when a larger share of businesses report insufficient demand as a factor that limits their decision to produce, this tends to be significantly associated with a disinflationary period. Conversely when fewer businesses declare that they face insufficient demand, inflation tends to rise.

We then focus on producer prices only and add some controls to refine the regression: real GDP growth, unemployment and lagged inflation. The results broadly confirm the previous observation, in the sense that shortages of materials and equipment are indeed the most significant factor associated with producer price dynamics, followed by lack of demand.

These results allow us to select these two indicators to proxy supply and demand shocks, as they are the two

4 Data collected by the Joint Harmonised EU Programme of Business and Consumer Surveys are available at https://ec.europa.eu/info/business-economy-euro/indicators-statistics/economic-databases/business-and-consumer-surveys_en.

main factors with a significant association with inflation trends, in particular when we look at producer prices (Table 2). For this reason, we use them as our best predictors of the impulses originating from a supply and from a demand shock.

We define the inflationary impulses originating from supply factors by calculating the number of standard deviations of the share of respondents signalling supply shortages in the most recent quarter with respect to the historical average.[5] The intuition behind this choice is that when the share of businesses complaining about shortages of materials and equipment is higher in historical comparison, we may expect prices to increase.

In the equation below, $SUP_{tz}$ indicates the inflationary supply impulse at time t and in sector $z$; $S_{tz}$ indicates the share of businesses reporting shortages of materials and equipment as the main factor hampering production at time t and in sector $z$; $AvS_{(t\text{-}1),z}$ refers to the historical average of this same share, between 1991 and the period preceding the latest observation (t-1); $StdS_{(t\text{-}1),z}$ refers to the standard deviation of the same share, between 1991 and the period preceding the latest observation (t-1):

$$SUP_{tz} = \frac{S_{tz} - AvS_{(t-1),z}}{StdS_{(t-1),z}}$$

Similarly, but in a symmetric way, we use the share of businesses reporting a lack of demand as a main factor hampering production, as a proxy for a demand shock. In this case, however, the indicator is negatively associated with producer price increases. Therefore, in order to proxy an inflationary demand shock, we need to take it with an inverted sign. We proxy inflationary impulses originating from demand factors by calculating the negative of the number of standard deviations of the share of respondents signalling lack of demand in the most recent quarter with respect to the same historical average. The intuition behind this choice is that when complaints about scarce demand decrease by historical standards, we may be facing an inflationary demand impulse.

Below, $DEM_{tz}$ indicates the inflationary demand impulse at time t and in sector $z$; $D_{tz}$ indicates the share of businesses reporting a lack of demand as the main factor hampering production at time t and in sector $z$; $AvD_{(t\text{-}1),z}$ refers to the historical average of this same share, between 1991 and the period preceding the latest observation (t-1); and $StdD_{(t\text{-}1),z}$ refers to the standard deviation of the same share, between 1991 and the period preceding the latest observation (t-1):

5 The time series on which we calculate historical average and the standard deviation starts from 1991Q1.

Table 2

**Producer price inflation in industry and factors limiting production**

| Dependent variable | Producer price inflation in industry | | |
|---|---|---|---|
| No factor limiting production | -0.01 (.03) | -0.03 (.04) | -0.01 (.01) |
| Lack of demand | -0.10 *** (.02) | -0.05 * (.02) | 0.01 (.01) |
| Shortages of materials and equipment | 0.30 *** (.06) | 0.24 *** (.05) | 0.08 *** (.02) |
| Shortages of labour | 0.01 (.03) | -0.03 (.04) | -0.01 (.01) |
| Financial constraints | 0.05 (.04) | 0.08 (.04) | -0.01 (.01) |
| Other factors (unspecified) | 0.03 (.03) | 0.02 (.03) | 0.02 * (.01) |
| Control: Unemployment rate | | -0.19 * (.09) | -0.03 (.03) |
| Control: Real GDP growth rate | | 0.31 ** (0.9) | 0.23 *** (.03) |
| Control: Lagged pp inflation at (t-1) | | | 0.79 *** (.01) |
| Constant | 2.74 (1.69) | 3.33 (2.01) | -0.21 (0.64) |
| Fixed effects | yes | Yes | yes |
| No. of observations | 2021 | 1983 | 1972 |
| $R^2$ | 0.23 | 0.23 | 0.75 |
| Adjusted $R^2$ | 0.23 | 0.23 | 0.74 |

Note: * p<0.05, ** p<0.01, *** p<0.001. Robust standard errors in parentheses.

Source: Author's own calculation.

$$DEM_{tz} = -\left(\frac{D_{tz} - AvD_{(t-1),z}}{StdD_{(t-1),z}}\right)$$

We then scale the two relative shares to the overall producer-price inflation rate in each sector. In the formula, the two shares are calculated on the basis of the year-on-year producer price inflation $PP_{tz}$ of sector $z$ at time t:

$$SUP_{tz}\,share = \frac{SUP_{tz}}{SUP_{tz} + DEM_{tz}} PP_{tz} \text{ and}$$

$$DEM_{tz}\,share = \frac{DEM_{tz}}{SUB_{tz} + DEM_{tz}} PP_{tz}$$

It may happen that in some quarters, the two impulses move in opposite directions: an inflationary supply impulse and a deflationary demand impulse (or vice versa). In these cases, the difference between the relative impulses equals the inflation rate.

## Decomposition of producer price inflation

The attribution of the total year-on-year inflation rate of producer prices in industry to the two supply and demand impulses, derived as explained above, allows for the decomposition of the inflationary shock and estimation of the relative importance of supply and demand factors, in each quarter. Figure 7 presents the results for total industry sectors in the euro area.

The current spike in producer price inflation for industry is unprecedented over the past 20 years. We can see that the recession of 2009 corresponded to a large deflationary shock mainly driven by a contraction in demand, which was followed by a period of relatively higher price pressure, mainly driven by supply constraints, as supply had to adjust to a recovery in demand. The series then shows another relatively small episode of falling prices in 2016, mainly due to deflationary supply shocks. The pandemic and related lockdown in 2020 caused another small episode of falling prices, initially driven by supply constraints (probably due to the initial shock) and later by falling demand (likely due to the containment measures).[6]

Nevertheless, none of these episodes is comparable in magnitude to the present inflationary shock. The increase in producer prices in industry in the euro area during the past four quarters is unprecedented. This decomposition approach allows us to say that 80% of the current shock is due to supply shortages and only 20% to demand boost. After being very weak during the pandemic, demand is now putting some pressure on prices; however the inflationary pressure coming from demand expansion is not exceptional in the historical perspective. In other words, while the demand pressure seems to be contributing to price pressures to some extent, the relevance of the supply shock is so big that it can be considered four times stronger than that of the demand.

## Sectoral detail

Manufacturing sectors are experiencing historically high pressure on producer prices. The most affected ones are energy-intensive industries, chemicals, wood, paper, rubber and plastics, and food (see Figure 8). The detail of these sectors shows that the unprecedented price pressure is fully linked to a rapidly deteriorating assessment of supply factors, suggesting that supply disruptions may be the main cause. The energy shock certainly explains this result. The energy market is very upstream in the production process, so any disruption, and in particular the huge ones currently happening, add strong pressure on the supply side of the euro area economy.

6 O'Brien et al. (2021) study the impact of the pandemic across inflation components in 2020 and find a dominant role for downward domestic and global demand effects, only partly offset by upward supply effects, which were strongest in the second quarter of 2020 and more prevalent in goods than in services. They claim, however, that the role of supply-side effects in particular is likely to be larger than usual for a number of inflation components.

Figure 7

**Decomposition of producer price inflation in supply and demand factors in the euro area**

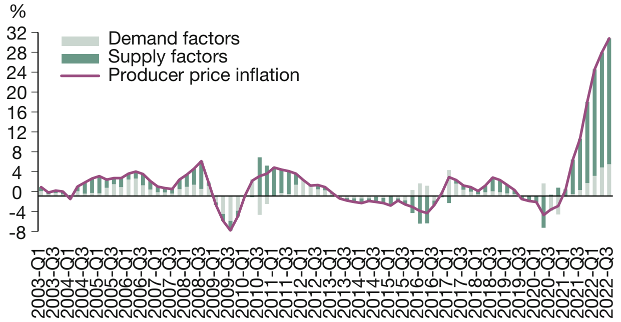

Note: The chart refers to total industry sectors; quarterly data.

Source: Author's elaboration.

Large sectors, such as the manufacturing of motor vehicles, pharmaceuticals and machineries, are clearly facing sizeable supply shortages that are driving up prices. The impulse originating from demand seems to be playing a moderate role in producer price inflation for all of these sectors. The manufacturing of coke and refined petroleum products is the sector experiencing the highest price pressure. This decomposition shows that price increases in this sector are almost entirely due to supply constraints.

A similar profile emerges in the case of the manufacturing of basic metals, the second most affected sector by price pressure. In this sector, though, the overall inflation rate seems to be decreasing in the second quarter of this year, amid easing supply pressures and increasing demand ones. The other major industrial sectors display similar patterns to the overall aggregate of industry, and supply factors account for at least 80% of the inflation shock in all of them.

## Country detail

We replicate the analysis at country level, for total industry, in order to understand to what extent the general profile defined for the euro area as a whole applies to member states in a similar way or if there are significant differences.

Countries differ, first of all, for the level of producer price inflation, with peaks of almost 40% year-on-year price increase in the Netherlands or Italy, and more moderate rates in Malta (4.3%) or Ireland (12.0%).

Nevertheless, our decomposition shows also some differences in terms of the underlying factors driving up producer

Figure 8

**Decomposition of sectoral producer price inflation in supply and demand factors**

in %

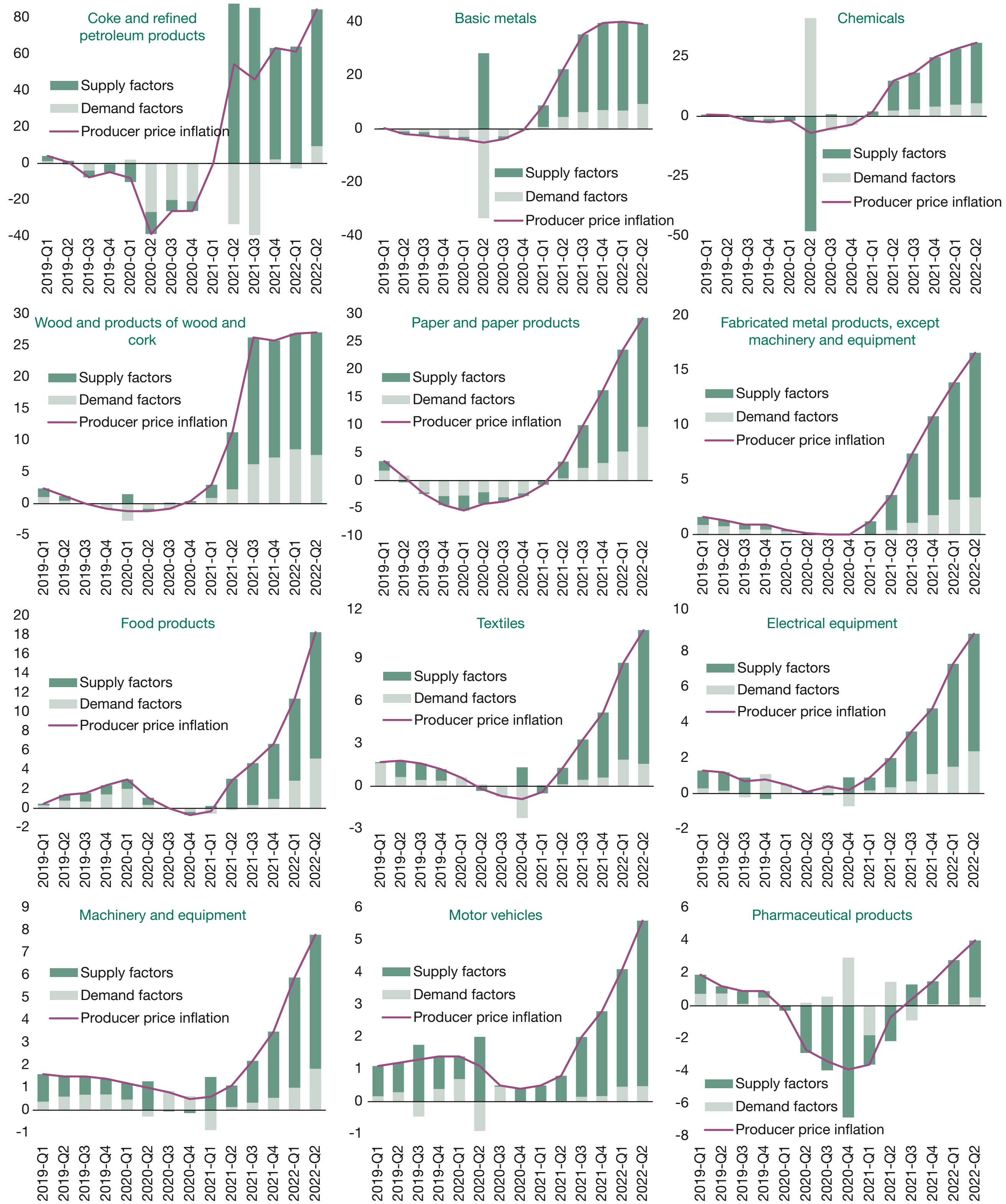

Note: The charts refer to the euro area; quarterly data.

Source: Author's elaboration.

Figure 9

**Decomposition of producer price inflation in supply and demand factors, selected countries**

in %

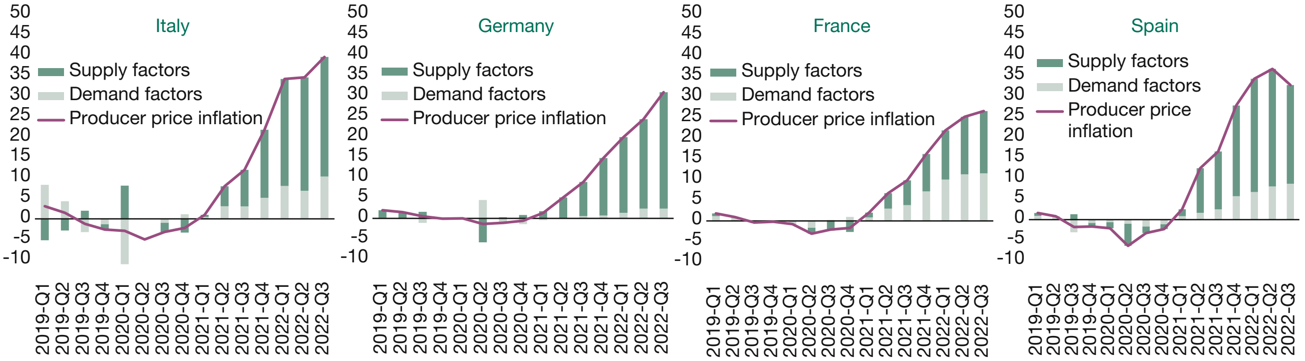

Note: The charts refer to total industry sectors; quarterly data.

Source: Author's elaboration.

prices in industry. Supply factors remain more important than demand factors in all countries; however, in some cases demand factors seem quite relevant, too. The analysis of the four largest economies of the euro area, presented in Figure 9, shows that in Germany, for instance, demand factors are almost irrelevant to explain the current inflationary pressure. In France, on the other hand, they account for almost half of the total pressure.

## Discussion

The recent volatility in price levels, driven by unprecedented global shocks, has spurred several theoretical and empirical contributions aiming at understanding the relative importance of supply and demand shocks in inflationary pressures (Lian and Freitag, 2021; O'Brien et al., 2021; Celasun et al., 2022; Mullin, 2022; Ruge-Murcia and Wolman, 2022; Shapiro, 2022b).

In the case of the United States, Shapiro (2022b) finds that supply factors are responsible for more than half of the recent high PCE inflation, reflecting supply constraints from disruptions related to the pandemic and the war in Ukraine. In the euro area, however, when looking at producer prices, we find that even more (at least 80%) are due to supply constraints. Such supply constraints depend mainly on the current energy crisis.

The implications of our results, based on businesses' perceptions of ongoing supply and demand dynamics, are consistent with the recent findings of the literature on this matter. Baqaee and Farhi (2022), for instance, find that negative sectoral supply shocks are stagflationary, whereas negative demand shocks are deflationary. Di Giovanni et al. (2022) apply Baqaee and Farhi's model to study the euro area inflation shock over the period 2020-2021 and find that foreign shocks and global supply chain bottlenecks played an outsized role relative to domestic aggregate demand shocks.

The observation that a historically large negative supply shock (inflationary) is taking place and being sustained over time is particularly important and worrying. Guerrieri et al. (2022), in fact, explain that shocks that reduce potential output in a sector of the economy, by reducing demand in other sectors, ultimately push aggregate activity below potential. This is more likely when the elasticity of substitution between sectors is relatively low, the intertemporal elasticity of substitution is relatively high, and markets are incomplete.

By raising prices and constraining economic activity, supply shocks carry high risks of entering a period of low growth. If this is what is happening in the euro area, as our partial analysis of the most affected industrial sectors seems to suggest, such dynamics may soon trigger a contraction in output. If this tendency becomes generalised to the whole economy, and if it is confirmed by actual output data, the downside risks to the economic outlook would be greater, with the unpleasant perspective of a recession.

The first line of defence against inflation, monetary policy, may face some difficulties in achieving its objective. Its effectiveness will be proportional to the extent that demand factors cause inflation. But if they are of limited importance in the present context, then, monetary policy needs other policies to support its operation. In particular, positive supply shocks are needed to compensate the negative supply shocks that reduce potential output. For this reason, in the present context, investment in repairing supply chains disruptions, in mitigating the energy shock and preventing future disruptions, is of the essence. That will certainly not be an easy task; on the one side, the energy transition will take time to fully materialise; on the other, new geopolitical tensions may interfere with the attempt to repair global supply chains and let them work as they did in the past. These challenges imply that part of the current supply constraints may not abate soon, thereby keeping some pressure on European industry.

Moreover, to the extent that the underlying balance between supply and demand factors differs across countries within the monetary union, a common monetary policy can hardly avoid the risk of divergences. The effectiveness of monetary policy in reining in inflation will be directly proportional to the relative importance of demand factors in driving price pressure. So, it can be expected to be more effective in reducing inflation in countries in which demand factors play a bigger role (France) than in countries in which their role is almost negligeable (Germany). In this second case, targeted investment will be crucial, not only to rebuild potential output, but also to avoid divergences.
